# Simulation effectiveness tool modified (SET-M): adaptation and validation for Brazil*

**DOI:** 10.1590/1518-8345.4282.3437

**Published:** 2021-06-28

**Authors:** Ellen Cristina Bergamasco, Diná de Almeida Lopes Monteiro da Cruz

**Affiliations:** 1 Faculdade Israelita de Ciências da Saúde Albert Einstein, Faculdade de Enfermagem, São Paulo, SP, Brazil.; 2 Universidade de São Paulo, Escola de Enfermagem, São Paulo, SP, Brazil.

**Keywords:** Simulation Technique, Validation Study, Effectiveness, Surveys and Questionnaires, Nursing Education, Simulation Training, Simulação, Estudo de Validação, Efetividade, Inquéritos e Questionários, Educação em Enfermagem, Treinamento por Simulação, Simulación, Estudio de Validación, Efectividad, Encuestas y Cuestionarios, Educación em Enfermería, Entrenamiento Simulado

## Abstract

**Objective:**

to adapt the Simulation Effectiveness Tool - Modified (SET-M) to Portuguese and to verify validity and reliability indexes.

**Method:**

methodological study using ISPOR, Confirmatory Factor Analysis, correlation between the adapted instrument/Simulation Design Scale - Student Version/Individual Practice Assessment and reliability (test-retest and internal consistency indexes). Convenience sample with a total of 435 Nursing undergraduate and graduate students.

**Results:**

Simulation Effectiveness Tool - Modified Brazilian Version obtained an average score between 2.36 to 2.94. The Confirmatory Factor Analysis had a factor load > 0.30 for 17 of the 19 items. Cronbach’s alpha ranged between 0.729 and 0.874. McDonald’s omega was 0.782. There was no correlation between Simulation Effectiveness Tool - Modified Brazilian Version and the Simulation Design or Individual Practical Assessment. There was a positive correlation between the Simulation Effectiveness Tool - Modified Brazilian Version and the participants’ age. The scores of the volunteers in the simulations were significantly higher than those of the observers in three domains.

**Conclusion:**

the SET-M Brazilian Version, maintaining the 19 items and four domains of the original scale, was made available for use in Brazil to evaluate the effectiveness of the simulation, recommending studies with different samples.

## Abstract (pt)

**Objetivo:**

adaptar para a língua portuguesa o *Simulation Effectiveness Tool - Modified* (SET-M) e verificar índices de validade e confiabilidade.

**Método:**

estudo metodológico. Utilizou-se o *ISPOR*, Análise Fatorial Confirmatória, correlação entre o instrumento adaptado/Escala de *Design* da Simulação – Versão Estudante/Avaliação Prática Individual e confiabilidade (teste-reteste e índices de consistência interna). Amostra de conveniência com 435 estudantes da Graduação em Enfermagem e Pós-Graduação.

**Resultados:**

o *Simulation Effectiveness Tool - Modified* Versão Brasileira obteve média de escores entre 2,36 a 2,94. A Análise Fatorial Confirmatória mostrou carga fatoral > 0,30 para 17 dos 19 itens. O alfa de Cronbach variou entre 0,729 e 0,874. O ômega de McDonald foi 0,782. Não houve correlação entre *Simulation Effectiveness Tool - Modified* Versão Brasileira e o *Design* da Simulação ou a Avaliação Prática Individual. Houve correlação positiva entre o *Simulation Effectiveness Tool - Modified* Versão Brasileira e a idade dos participantes. Os escores dos voluntários nas simulações foram significativamente mais altos que os dos observadores em três domínios.

**Conclusão:**

o SET-M Versão Brasileira, mantendo os 19 itens e quatro domínios da escala original, ficou disponibilizado para ser usado no Brasil para avaliar a efetividade da simulação, recomendando-se estudos com amostras diferentes.

## Abstract (es)

**Objetivo:**

adaptar el *Simulation Effectiveness Tool - Modified (SET-M)* al portugués y verificar los índices de validez y confiabilidad.

**Método:**

estudio metodológico. En esta etapa de la investigación se utilizó el *ISPOR*, Análisis Factorial Confirmatorio, correlación entre el instrumento adaptado/Escala de *Design* de la Simulación - Versión Estudiante/Evaluación Práctica Individual y confiabilidad (test-retest e índices de consistencia interna). Muestra de conveniencia con 435 estudiantes de licenciatura en Enfermería y del posgrado.

**Resultados:**

el *Simulation Effectiveness Tool – Modified* - Versión Brasileña obtuvo un puntaje promedio entre 2,36 a 2,94; el Análisis Factorial Confirmatorio mostró una carga factorial > 0,30 para 17 de los 19 ítems. El *alpha* de Cronbach osciló entre 0,729 y 0,874; El omega de McDonald fue 0,782. No hubo correlación entre *Simulation Effectiveness Tool – Modified* Versión Brasileña y el *Design* de la Simulación o la Evaluación Práctica Individual. Hubo una correlación positiva entre el *Simulation Effectiveness Tool – Modified* Versión Brasileña y la edad de los participantes. Los puntajes de los voluntarios en las simulaciones fueron significativamente más altos que las de los observadores en tres dominios.

**Conclusión:**

el SET-M Versión Brasileña, manteniendo los 19 ítems y cuatro dominios de la escala original, se puso a disposición para su uso en Brasil para evaluar la efectividad de la simulación, recomendándose estudios con diferentes muestras.

## Introduction

The training of nurses is an increasing challenge since, in addition to technical skills, it is also necessary to develop attitudinal and managerial aspects. Active strategies such as simulation can provide argumentation, reflection and practice. Thus, simulation is a pedagogical approach that has been growing^(^ 1 ^)^.

The principle of simulation is the construction of fictitious situations, like real ones, with the aim of providing the participant with the possibility of practicing their skills safely. It allows students to develop their skills based on situations close to real ones, learning the best way to assist a patient when something similar happens in a real situation^(^ 2 ^)^.

Many studies have been published on nursing simulation; however, few address the effectiveness of this strategy and its effects on care practice. The effectiveness of the simulation seeks to assess the students’ ability to transfer knowledge to the context of the real world^(^ 3 ^)^, and may be related to the students’ self-perception about their learning and self-confidence. An instrument capable of evaluating these concepts can help professors in the elaboration and conduction of best practices in simulation^(^ 4 ^)^, in addition to the production of evidence related to this strategy.

In Brazil, there are no instruments available to evaluate the effectiveness of the simulation, but the literature has the Simulation Effectiveness Tool - Modified (SET-M)^(^ 5 ^)^. SET-M is an enhanced version of the Simulation Effectiveness Tool (SET)^(^ 4 ^)^, which was developed in 2012 with the aim of evaluating students’ perception of the simulation experience^(^ 4 ^)^. Linked to the creation of the Medical Education Technologies Incorporated (METI®) program for teaching nursing, this one and other instruments were part of the Program for Nursing Curriculum Integration (PNCI), which was intended to build a script of simulated clinical experiences to serve as a recommendation for instructional documentation and to integrate simulation activities into the nursing curriculum in the United States^(^ 4 ^)^. The initial version of the instrument consisted of a total of 20 items, with a 5-point Likert scale. This version was applied to students and after evaluating the psychometric properties, it resulted in an instrument with 13 items, and a 3-point response scale^(^ 4 ^)^.

In 2015, this instrument was revised and was named Simulation Effectiveness Tool - Modified (SET-M)^(^ 5 ^)^, with a total of 19 items, using a 3-point response scale. The items of this instrument are divided into four domains: Pre-briefing - items 1 and 2; Learning - items 3 to 8; Trust - items 9 to 14; and Debriefing - items 15 to 19.

This instrument has been used for simulation, but it could not be applied to the Brazilian context since it was not available for the Portuguese language. This study aimed to adapt the SET-M for Brazil, to evaluate the validity of content, construct, criterion, reliability, in addition to verify the associations between SET-M adapted for Brazil and age, year of the Undergraduate course, time of training of the participant and participation as a volunteer or observer in the scenario.

## Method

This is a psychometric study of SET-M adaptation and validation, with quantitative approach and cross-section. It was performed in three stages: Stage I - Translation, adaptation and evaluation of content validity (Following the steps recommended by the International Society for Pharmacoeconomics and Outcomes Research - Translation and Cultural Adaptation - ISPOR TCA Task Force)^(^ 6 ^)^; Stage II - Verification of construct validity (Factor Analysis and estimates of reliability and stability); Stage III - Verification of the criterion validity.

A total of 435 people participated in Stage II (214 Nursing undergraduate students and 221 graduate students at Albert Einstein Israel University of Health Sciences - FICSAE); they were asked to answer the SET-M Brazilian Version immediately after participating in a simulated activity. These simulations took place between March and July 2019. Stage III was carried out with a total of 21 Nursing undergraduate students at FICSAE after the Venous Access Care Workshop. Figure 1 shows Stages II and III considering the type of validity, the analysis performed and the number of participants.

**Figure 1:** Stage II and III: validity types, analysis and number of participants. São Paulo, SP, Brazil, 2020

Stage	Analysis	Number of participants (n)
II - Construct Validity	Confirmatory Factor Analysis	240
	Reliability - Internal consistency of domains - Cronbach's alpha	
	Reliability - Internal Instrument Consistency - McDonald's Omega	
	Stability - Test and Retest Method - Intraclass Correlation Coefficient	34
	Correlations between variables - Kendall's correlation coefficient - Age and training time (Pearson's Correlation Coefficient) - Year of the undergraduate course, type of participation in the simulated activity and number of participations as an observer or volunteer	435
III - Criterion Validity	Spearman's Correlation Test - Correlation between the scores of SET-M Brazilian Version and - Simulation Design Scale - Student Version (SDS-SV) - Individual Practical Assessment (IPA) held at the Venous Access Care Workshop.	21

To verify the construct validity, a Confirmatory Factor Analysis (CFA) was performed. The model tested followed what was described in the development of the original instrument with four domains. The CFA method was robust weighted least squares estimation, with the model fit being verified by the ratio between the chi-square (χ^2^) which was 1, with p<0.001, and by the CFI (Comparative Fit Index) adjustment indexes of 0.929, TLI (Tucker Lewis Index) of 0.917, RMSEA (Root-Mean-Square Error of Approximation) of 0.047 and SRMR (Standardized Root Mean Square Residual) of 0.060. Despite the 435 instruments, we used only the questionnaires that had all items filled out (240), since no data were entered into questionnaires with blank items.

To assess the internal consistency, we verified the values of Cronbach’s alpha for each domain, since the McDonald’s omega evaluated the internal consistency among all items on the instrument. Stability was assessed by the test-retest method using the Intraclass Correlation Coefficient comparing the responses of SET-M on the day of the simulation and the responses obtained 30 days later.

The scores in the SET-M domains were studied for possible correlations with the following variables: age and training time (Pearson’s Correlation Coefficient); year of the undergraduate course, type of participation in the simulated activity and number of participations as an observer or volunteer (Correlation Coefficient by Kendall posts).

Criterion validity used the Spearman Correlation Test to assess the correlation between the scores of the SET-M Brazilian Version and the Simulation Design Scale, Student Version (SDS-SV), in addition to the Individual Practical Assessment (IPA) performed at Venous Access Care Workshop. It consisted of a dialogue expository class (duration of 30 minutes), followed by a simulation activity (scenario with standardized patient and debriefing - total duration of 30 minutes). After the simulation, the participants performed the IPA. Each participant was observed by two independent evaluators who used a checklist to analyze the student’s practical performance. We calculated the average of correct answers between the observers and the Intraclass Correlation Coefficient (Kappa Coefficient) of the scores and it was 0.40 (F 2.36; p=0.028; 95%CI -0.01 - 0.70).

The project started after approval by the Research Ethics Committee under number CAAE: 05798818 0 0000 0071. The author of the original instrument authorized the SET-M translation and adaptation process.

## Results

Stage I of translation and adaptation of SET-M followed the steps proposed by the ISPOR TCA Task Force^(^ 6 ^)^. Steps 1 (preparation) and 2 (translation) resulted in Translated Version 1 (TV1) and Translated Version 2 (TV2). From these versions, reconciliation was performed (Step 3), resulting in Version TV1-2. Step 4 consisted of sending the TV1-2 for back-translation by two professionals that produced the Back Translated Version 1 (BTV1) and Back Translated Version 2 (VRT2), both of them were sent to the author of the instrument and considered for the BackTranslation Review (Step 5). The author made observations about the words used in some items; an important recommendation was that we use the original name of the scale including “Brazilian Portuguese Version”. So, we chose the expression “Brazilian Version”. After this step, a meeting was held with the expert committee in which all versions and comments sent by the author were analyzed. In this stage, the Content Validity was carried out and, at the end, we had the TV3 of the instrument. Step 6 was not performed since it does not apply to our study. Step 7 consisted of the application of the TV3 by performing cognitive debriefing in which a total of 9 students participated.

In Step 8, the cognitive debriefing was reviewed, and the instrument adjustments were finalized. The main adjustments were insert a brief explanation next to the words “Pre-Briefing”, “Scenario” and “Debriefing”. We asked about the application of item 6 only for those who volunteered because only those who were within the scenario may have felt empowered to make the decision at the time of the change. In addition, the word “felt” gives the understand that “I felt empowered in the specific scenario”. Thus, the suggestion was to change it to “I feel”, in the present tense, meaning that “from now on, after the simulation, I feel empowered to attend to cases like this”.

In the participants’ perception of cognitive debriefing, the eighth item of the instrument applies only to those who participated as a volunteer, justifying that “practicing” means doing something, since the observers were unable to practice. The suggestion was to replace the word “practice” with “reflect”, so it can be applicable for volunteers and observers. We chose to maintain the original item (8) and insert an extra one, as a modification of that item, considering those who were observers in the simulation (extra item 14 “I had the opportunity to reflect on my decision-making skills”). In the validation stage of this study, we applied the instrument with two items (8 and extra item 14).

Item 16 raised a lot of discussion and it was decided to keep it as “Debriefing allowed me to first verbalize my feelings and then focus on the scenario”. Regarding the question at the end of the instrument, the expression “simulated clinical experience” was questioned since in the context of teaching in Brazilian institutions, we use clinical and non-clinical scenarios such as those that deal with feedback or issues of communication and interpersonal relationships. Considering that we would like to apply the instrument in this type of scenario, after talking between the researchers and the author of the instrument, we chose to exclude the word “clinic”.

After adjusting the instrument, we obtained TV4, containing a total of 20 items (19 items translated and adapted, plus the extra item 14 that was inserted by the researchers during step 8) and a discursive question in which the participants can add something they want to say about the simulated experience. Step 9 was carried out by revising the Portuguese language, but there were no changes in the items. The final report (Step 10) corresponds to the results found.

Then, the TV4 was applied to the participants of this study in several simulations, so that the evidence of construct validity and criteria of the Simulation Effectiveness Tool - Modified (SET-M) Brazilian Version could be evaluated.

Among the 214 undergraduate students, 91% were female and the average age was 27.8 years old; while graduate students had an average of 28.5 years old and 90% were female. Of the total of 435 participants, 94% said they had previously participated in some scenario and 52% reported participation as a volunteer, 25% in one scenario, 14% in two scenarios, 4% in three scenarios and 9% in four or more scenarios.

Table 1 shows that items from Domains 1, 2 and 3 have a satisfactory factor load and Domain 4 has two items with very low load (<0.30).

**Table 1:** SET-M Confirmatory Factor Analysis Statistics Brazilian Version, considering the 4 domains of the original scale (N=240). São Paulo, SP, Brazil, 2020

Latent Variable	Factor Load	Standard error	Estimate divided by standard error	p
Domain 1 - Pre-briefing				
Item 1. Prebriefing increased my confidence.	0.527	0.042	12.475	< 0.001
Item 2. Prebriefing was beneficial for my learning.	0.388	0.048	8.053	< 0.001
Domain 2 - Learning				
Item 3. I am better prepared to respond to changes in my patient's condition.	0.382	0.033	11.618	< 0.001
Item 4. I developed a better understanding of the pathophysiology.	0.494	0.033	15.180	< 0.001
Item 5. I am more confident of my nursing assessment skills.	0.425	0.033	12.875	< 0.001
Item 6. I felt empowered to make clinical decisions.	0.494	0.037	13.187	< 0.001
Item 7. I developed a better understanding of medications. (Leave blank if no medications in scenario).	0.445	0.036	12.498	< 0.001
Item 8. I had the opportunity to practice my clinical decision making skills.	0.449	0.039	11.404	< 0.001
Domain 3 - Trust				
Item 9. I am more confident in my ability to prioritize care and interventions.	0.408	0.029	14.060	< 0.001
Item 10. I am more confident in communicating with my patient.	0.348	0.025	13.724	< 0.001
Item 11. I am more confident in my ability to teach patients about their illness and interventions.	0.426	0.033	12.748	< 0.001
Item 12. I am more confident in my ability to report information to health care team.	0.398	0.028	14.199	< 0.001
Item 13. I am more confident in providing interventions that foster patient safety.	0.406	0.025	16.320	< 0.001
Item 14. I am more confident in using evidence-based practice to provide nursing care.	0.401	0.033	12.003	< 0.001
Domain 4 - Debriefing				
Item 15. Debriefing contributed to my learning.	0.116	0.031	3.707	< 0.001
Item 16. Debriefing allowed me to verbalize my feelings before focusing on the scenario.	0.349	0.050	6.996	< 0.001
Item 17. Debriefing was valuable in helping me improve my clinical judgment.	0.311	0.033	9.348	< 0.001
Item 18. Debriefing provided opportunities to self-reflect on my performance during simulation.	0.385	0.046	8.376	< 0.001
Item 19. Debriefing was a constructive evaluation of the simulation.	0.216	0.041	5.207	< 0.001

Table 2 shows that Domain 2 has a very high covariance with Domain 3.

The results of Cronbach’s alpha for the four domains obtained a satisfactory result (>0.7). Table 3 shows that they worsen when excluding any item from the domain.

**Table 2:** Covariance among the four domains of the SET-M Brazilian version, considering the domains of the original scale. São Paulo, SP, Brazil, 2020

Covariance	Load	Standard error	Estimate divided by standard error	p
Domain 1 - Pre-briefing				
Domain 2 - Learning	0.643	0.062	10.451	< 0.001
Domain 3 - Trust	0.577	0.061	9.448	< 0.001
Domain 4 - Debriefing	0.320	0.100	3.191	0,001
Domain 2 - Learning				
Domain 3 - Trust	0.922	0.024	39.202	< 0.001
Domain 4 - Debriefing	0.575	0.060	9.574	< 0.001
Domain 3 - Trust				
Domain 4 - Debriefing	0.617	0.062	9.875	< 0.001

**Table 3:** Cronbach's alpha of the domains of SET-M Brazilian version and alpha if each item is excluded. São Paulo, SP, Brazil, 2020

Item	Cronbach's alpha if the item is deleted	Correlation of the item with the total	Correlation of the item with the corrected total	Final correlation if the item is deleted
Domain 1 - Pre-briefing - Cronbach's alpha 0.792				
Item 1	0.664	0.926	0.743	Not applicable
Item 2	0.664	0.897	0.743	Not applicable
Domain 2 - Learning - Cronbach's alpha 0.823				
Item 3	0.533	0.688	0.599	0.533
Item 4	0.612	0.748	0.678	0.612
Item 5	0.595	0.727	0.656	0.595
Item 6	0.606	0.743	0.67	0.606
Item 7	0.678	0.765	0.75	0.678
Item 8	0.527	0.686	0.588	0.527
Domain 3 - Cronbach's alpha 0.874				
Item 9	0.635	0.750	0.677	0.635
Item 10	0.608	0.732	0.652	0.608
Item 11	0.659	0.778	0.703	0.659
Item 12	0.744	0.834	0.803	0.744
Item 13	0.739	0.826	0.793	0.739
Item 14	0.686	0.789	0.739	0.686
Domain 4 - Debriefing- Cronbach's alpha 0.758				
Item 15	0.448	0.580	0.546	0.448
Item 16	0.530	0.784	0.588	0.530
Item 17	0.523	0.702	0.579	0.523
Item 18	0.644	0.800	0.740	0.644
Item 19	0.617	0.741	0.730	0.617

## Discussion

The name of the instrument remained Simulation Effecctiveness Tool - Modified (SET-M) Brazilian version and considered 19 items, divided into four domains. During the translation and adaptation stages, we insert a brief explanation of these terms beside the terms “Pre-briefing”, “Scenario” and “Debriefing” to ensure that participants know what stage we are referring to. The pre-briefing corresponds to the guidance given immediately before the start of the scenario in which preparatory instructions are provided to participants and guidance on the scenario^(^ 7 ^)^. It is worth noting that even if the scenario takes place in a room/laboratory where only volunteers receive guidance on the mannequin and equipment, guidance on scenario objectives and full case is provided for all participants (volunteers and observers).

Thus, at the end of the translation and adaptation, the SET-M Brazilian Version had a total of 20 items, 19 from the original instrument and the extra item 14 was modification of item 8. For the construct validity, the instrument was applied to Nursing undergraduate and graduate students, since studies using SET-M have been carried out with both populations.

The frequency of response was greater than 52% for “Totally Agree” on most items. The agreement obtained in this study was lower than in the validation study of the original scale (greater than 67.6% for all items)^(^ 5 ^)^. When we grouped the responses “Totally Agree” and “Partially Agree”, only one item from SET-M did not find agreement equal to or greater than 91%.

Very similar results were obtained in a study that used SET for nursing students in simulation on mental health, in which 12 of the 13 items had total/partial agreement equal to or greater than 91%^(^ 8 ^)^. A study that used SET-M and addressed patient safety pointed out that 94% of the participants totally or partially agree that the pre-briefing was effective, while 89% totally or partially agree with the effectiveness of the Debriefing experienced in the simulation^(^ 9 ^)^.

Studies using SET have found answers with high agreement, and if we consider all of them, we can say that most participants agree with almost all items. Although this is a good result, it can direct our assessment to two different things: most simulation activities are effective and allow the participant to feel confident in their learning and skills of caring for their patient; and eventually the instrument may not be able to measure the full extent of the phenomenon. So, we suggest that multicenter studies be carried out using the SET-M Brazilian Version, to observe whether the instrument can measure a greater amplitude of the phenomenon. Another possibility would be to use the Rasch Analysis model.

Item 7 obtained 40% blank responses in the application of SET-M Brazilian Version. This is not a worrying fact, since the item may not be answered if the scenario does not involve activities related to medications. However, the original study^(^ 5 ^)^ describes that only 8.3% of the participants did not respond to this item. This result was probably due to the different characteristics of the simulations carried out in the studies. In our study, we applied the instrument after scenarios to discuss psychomotor and attitudinal skills. Thus, we understand that some simulations did not address the use of medications, such as the nasoenteral tube care scenario. The original study did not describe the scenarios used, reporting that students who attended medical-surgical nursing participated in the study, which leads us to believe that, most likely, the scenarios have addressed the use of some medication.

Thus, we can think that probably SET-M is not a very appropriate instrument for situations in which the main focus of the scenario is attitudinal, such as a proposed scenario to discuss the stages of feedback, or communication and negotiation skills. In our study we considered all scenarios, since our simulation programs built for Undergraduate or Graduate Studies are mixed, that is, they have scenarios to discuss psychomotor skills (medication administration, nasoenteral tube passage, cardiac arrest identification and Cardio-Pulmonary Resuscitation, among others), and attitudinal ones (behaviors for adverse events due to care errors and clinical decision-making, home care guidelines for family members of long-term patients, among others). We emphasize that other studies must be carried out to verify the effectiveness of simulations that primarily discuss attitudinal aspects, or that compare the use of SET-M in different types of scenarios, analyzing the behavior of item 7.

In our study, the average score obtained for each item of the instrument ranged from 2.36 to 2.94 and the items with the highest averages are in the Debriefing domain. The mean scores obtained in the validation study of the original scale were higher, with values between 2.71 and 2.90^(^ 5 ^)^. We understand that the items in the Debriefing domain are those that obtain the highest score averages, reinforcing the idea that these items have little response variability and that most participants perceive this step as very effective during the simulation. Again, we state that the simulations can have their Debriefing sessions conducted exceptionally, or the items that evaluate this stage are not sensitive enough to identify variations in response. Thus, we reaffirm the need for studies to assess the results of SET-M and to use instruments to assess Debriefing, making a comparison between these results. In addition, the use of the Rasch Model can also contribute.

The item-to-item correlation matrix varied between 0.27 and 0.67. When considering the correlations of the items in each domain, we observed a moderate correlation among the items in the Pre-briefing, Learning and Trust (values between 0.33 and 0.67), while in the Debriefing domain, the correlation between items 15, 16 and 17 assume values less than 0.30, but above 0.20. In the study published on the psychometric properties of the original SET-M, no correlation was excessively high (greater than 0.80), since some were less than 0.3, but none was less than 0.2 (5). Although we do not have access to the correlation matrix of this study, in general terms, the results are like those found with the SET-M Brazilian Version.

The correlations presented between extra item 14 and the other ones are moderate, however, of greater interest is the correlation of this item with item 8, which was weak (0.31). Thus, probably the two items (8 and 14 extra item) do not deal with the same part of the construct. If we consider that item 8 originated the extra item 14, being built with the possibility of replacing it, we expected this correlation to be quite strong, although this has not happened. This finding reinforced the idea that the items do not measure the same thing, which led us to the decision to keep the instrument with the original items. Then, we performed the Confirmatory Factor Analysis and the reliability assessment without the extra item 14.

In the Confirmatory Factor Analysis, all items in the Pre-briefing, Learning and Trust domains had good factor load (Table 1). Meanwhile, items 15 and 19 of the Debriefing domain had a factor load less than 0.30. It is important to remember that both items had little response variability, and, for factor analysis, the greater the variability, the more the item indicates that it is measuring what it wants to measure^(^ 10 ^)^, that is, the more the item is sensitive to discriminate the part of the construct to which it refers. Despite the results of items 15 and 19 in the CFA, as well as in the construct validation of the original instrument, we chose to maintain these items and test the importance of further studies to better assess their performance.

The covariance of the domains showed that Learning and Trust had a result of 0.922 (Table 2), which may mean that these domains overlap or deal with the same part of the construct. The active learning process can increase students’ satisfaction and self-confidence in conducting the simulated experience, indicating that educators should provide, whenever possible, opportunities for active student participation^(^ 11 ^)^. Debriefing methods improve psychomotor skills, self-confidence and students’ satisfaction, however, there is no evidence of superiority between the two modalities. Thus, it can be said that debriefing plays a fundamental role in learning, with a consequent improvement in satisfaction and self-confidence^(^ 12 ^)^.

Cronbach’s alpha (Table 3) has satisfactory indexes in the four domains: Pre-briefing (0.792), Learning (0.823), Confidence (0.874) and Debriefing (0.758). However, the original scale had higher rates: 0.833, 0.852, 0.913 and 0.908^(^ 5 ^)^. Eventually, these differences may be due to the sample size: a total of 435 participants in our study and 1288 in the original one.

The Intraclass Correlation Coefficient among the results of the retest test showed that the Pre-briefing and Learning domains have reasonable stability, respectively 0.427 (p<0.001) and 0.595 (p<0.001), while the Confidence domain has low stability, 0.155 (p=0.001), and the Debriefing domain did not find statistical significance (-0.032; p<0.746). These results may be related to the sample size or the time between the first and the second response (established in our study in 30 days). In general, it is recommended that the sample has at least 50 subjects^(^ 13 ^)^ and the time interval between checks is 10 to 14 days^(^ 14 ^)^. In planning the study, these recommendations were not considered, limiting the interpretation of the results.

The correlation between the SET-M Brazilian version scores and age was positive and weak. The results suggest that, the older the age, the better the perception of effectiveness of the simulation for learning. There was no association between the time since graduation and the scores obtained on the instrument.

The Kendall Correlation Coefficient showed that there was a weak and negative correlation (-0.163) between the year of graduation and the Pre-briefing domain (p=0.005), with no correlation with the other domains. This result suggests that, as the pre-briefing is more advanced in the program, the student is judged to be less effective. Possibly, students see very similar content in the pre-briefing among the various simulations, which, during the program, is less valued. Studies with multivariate analyzes are recommended to confirm and help clarify this result.

The tests of association between the scores of the SET-M Brazilian Version and the type of participation (volunteer or observer) showed that in all domains the scores of the volunteers were higher with statistically significant associations in three domains: Learning, Trust and Debriefing (p<0.001). The number of activities as a volunteer correlated weakly and positively with scores from the SET-M Brazilian Version in the Learning, Trust and Debriefing domains. For the Learning domain, the correlation was 0.219 (p<0.001), while in the Confidence domain it was 0.233 (p<0.001), and 0.176 (p<0.001) for the Debriefing domain. The number of participations as observers do not cause a difference in the perception scores of simulation effectiveness.

The results of the association tests allow us to state that those who participate as volunteers perceive greater effectiveness of the simulation than the observers in the Learning, Trust and Debriefing domains. It also allows us to state that the greater the number of participations as volunteers, the greater the perception of effectiveness of the simulation in the same domains.

Although the volunteers in our study assigned higher simulation effectiveness scores, all participants (volunteers and observers) assigned high scores with averages between 2.44 and 2.80 for observers, and 2.62 to 2.89 for volunteers. Thus, we can say that both perceived the experience of simulation as effective in their learning.

A literature review looking for evidence that evaluates observational learning methods and practical participation in the scenario discussed the data found in nine studies, five of which state that the observers’ learning results are as good or better than those that volunteered on the stage. The review concludes that learning results are closely related to the use of guides for directed observation (checklist) and the satisfaction of students who are in the role of the observer^(^ 15 ^)^.

These results support ours, considering that the simulation is effective for both observers and volunteers. However, they refute our result if we consider that there is a parallel between the perception of effectiveness and the tests of knowledge. In our investigation, the volunteers perceived greater effectiveness of the simulation in relation to the observers, although this should not be an argument for not using the simulation with observers, since in both types of participation the effectiveness is evidenced. The notes of the review study conclude that the learning results are related to the student’s involvement in learning, whether by actively participating in the simulation or being an active observer^(^ 15 ^)^.

A study carried out with a total of 251 pharmacy students, in which 130 were observers and 121 were volunteers in an interprofessional simulation, concluded that both observation and active participation can increase self-reported competence with regard to interprofessional collaboration^(^ 16 ^)^; thus, it can be said that, for both the volunteer and the observer, the reflections after the scenarios promote learning and contribute to the learners believing more in their abilities^(^ 17 ^-^ 18 ^)^.

A study with a total of 262 nursing and medical students showed that both observers and participants obtained similar results in three of the six predefined results, although the qualitative data highlighted the importance of the student participating in different functions, training repeatedly and discussing interprofessionally. In addition, the authors conclude that observing a simulation can be of great value for learning, but that the practice is still passed over by students, legitimizing the role of the observer, as long as the students also have the opportunity to practice^(^ 19 ^)^.

Thus, there are studies that claim that observing a simulation is as effective as actively participating in the scenario. In our study, we found that the simulation was effective for all participants, but the volunteers assigned statistically higher scores than the observers in the Learning, Trust and Debriefing domains. The results in which the volunteers attributed higher effectiveness scores using SET-M are original and suggest that other studies explore variables that can explain them. As an example, it is possible that there are other variables related to learning that favor the student to volunteer in the simulation scenarios. In this case, studies that explore how and why students volunteer for the scenarios would be interesting.

The criterion validity correlated the scores of the SET-M Brazilian Version with the scores of the SDS-SV and the IPA. It seemed reasonable to us to consider that a simulation evaluated with high Design scores (SDS-SV) would also have high effectiveness scores, however, Spearman’s correlation tests pointed out that there was no correlation between them. These results may be related to the sample number (n=21) or to the possibility that, contrary to our assumptions, SDS-SV deals with concepts that are not closely interconnected with SET-M. These results of no correlation should be considered with caution, especially due to the sample size. Therefore, we cannot claim that the two phenomena are not associated. In any case, it is also necessary to consider the possibility that the SDS-SV may not be a good instrument to carry out the SET-M criterion validity. Thus, we suggest that studies using SET-M, SDS-SV and other instruments be carried out with larger populations to assess criterion validity.

Individuals’ perception of their knowledge is not necessarily a reliable indicator of improvement, which indicates the need for objective tests^(^ 20 ^)^. In our study, we compared the SET-M scores with the scores of an individual practical assessment. The results showed that 76% of the participants correctly answered a total of 12 or 13 items of the assessment instrument, and the IPA scores did not have a statistically significant correlation. However, our sample was small, which does not allow us to state that there is not a correlation between the two phenomena. Thus, we suggest that studies be carried out with a larger number of participants.

Both the application of SET-M and the IPA did not have much variability, which may reinforce the idea that SET-M may not be a sensitive instrument to assess small changes in the perception of effectiveness of the simulation. This idea can also be replicated for the practical assessment, in which the items eventually fail to distinguish small differences in performance in the assessment.

In the search for investigations that used the SET, we found studies that added up all the items of the score, obtaining only one score for the assessment of effectiveness^(^ 21 ^)^; in addition, we also found studies with SET-M that added the scores of items in the Learning and Trust domains^(^ 9 ^)^ or even reduced the response categories, bringing together the categories “totally agree” and “partially agree” of the Likert-type scale. Thus, we consider suggesting to the authors of the original SET-M that they make recommendations on how to calculate the instrument’s score and how to present it in the publications of the studies that apply it, as this would help in the integration of knowledge about the effectiveness of the simulation using SET- M.

This study contributes to nursing education, as it provides an instrument that assesses the students’ perception regarding the effectiveness of the simulation. The limitations found are related to the sample size and the fact that the data were collected in a single educational institution. In this study, it was also not verified whether the type of simulation, in terms of focus (psychomotor or attitudinal skills or both), is associated with the properties of SET-M. Further investigations are needed to overcome this limitation of the study reported here.

## Conclusion

This study makes SET-M Brazilian Version available for use in Brazil, maintaining the total of 19 items and four domains of the original scale. The analyzes to verify the criterion validity of the SET-M Brazilian version were inconclusive, requiring studies with larger samples. The associations between the SET-M and the SDS-SV scores were not statistically significant, as were the associations between the SET-M and the IPA. The four domains had satisfactory reliability indexes (Cronbach’s alpha) and the MacDonald’s omega test had good reliability indexes for the scale. The items in the Pre-briefing, Learning and Trust domains have reasonable or low stability, however, the sample was small and the time for the retest could be shorter.

There was a correlation between participation as a volunteer and higher SET-M scores in the Learning, Trust and Debriefing domains; and weak correlations of the instrument with the participant’s age; there was no correlation with training time. The correlation was weak between the number of participations as a volunteer and the scores in the Learning, Trust and Debriefing domains; we found a weak and negative correlation between the graduation year and the Pre-briefing domain.

Simulation is a strategy that needs planning and teaching dedication. Thus, evaluating its effectiveness can be a stimulus point for educators to feel engaged in elaborating a scenario and conducting a simulation with quality and rigor. Having SET-M to evaluate the effectiveness of this teaching-learning strategy will allow obtaining empirical data to support decisions on the use of simulation.
